# Community SARS-CoV-2 seroprevalence before and after the second wave of SARS-CoV-2 infection in Harare, Zimbabwe

**DOI:** 10.1016/j.eclinm.2021.101172

**Published:** 2021-10-24

**Authors:** Arun Fryatt, Victoria Simms, Tsitsi Bandason, Nicol Redzo, Ioana D. Olaru, Chiratidzo E Ndhlovu, Hilda Mujuru, Simbarashe Rusakaniko, Michael Hoelscher, Raquel Rubio-Acero, Ivana Paunovic, Andreas Wieser, Prosper Chonzi, Kudzai Masunda, Rashida A Ferrand, Katharina Kranzer

**Affiliations:** a Biomedical Research and Training Institute, Harare, Zimbabwe; b MRC International Statistics and Epidemiology Group, Department of Infectious Disease Epidemiology, Faculty of Epidemiology and Population Health, London School of Hygiene & Tropical Medicine, London, UK; c Clinical Research Department, Faculty of Infectious and Tropical Diseases, London School of Hygiene & Tropical Medicine, London, UK; d Internal Medicine Unit, University of Zimbabwe Faculty of Medicine and Health Sciences, Harare, Zimbabwe; e Department of Paediatrics and Child Health, University of Zimbabwe Faculty of Medicine and Health Sciences, Harare, Zimbabwe; f Department of Community Medicine, Faculty of Medicine and Health Sciences, University of Zimbabwe, Harare, Zimbabwe; g Division of Infectious Diseases and Tropical Medicine, Medical Center of the University of Munich, Munich, Germany; h German Center for Infection Research (DZIF), Partner Site Munich, Munich, Germany; i Harare City Health, Harare, Zimbabwe

**Keywords:** SARS-CoV-2 seroprevalence, Zimbabwe

## Abstract

**Background:**

By the end of July 2021 Zimbabwe, has reported over 100,000 SARS-CoV-2 infections. The true number of SARS-CoV-2 infections is likely to be much higher. We conducted a seroprevalence survey to estimate the prevalence of past SARS-CoV-2 in three high-density communities in Harare, Zimbabwe before and after the second wave of SARS-CoV-2.

**Methods:**

Between November 2020 and April 2021 we conducted a cross-sectional study of randomly selected households in three high-density communities (Budiriro, Highfield and Mbare) in Harare. Consenting participants answered a questionnaire and a dried blood spot sample was taken. Samples were tested for anti-SARS-CoV-2 nucleocapsid antibodies using the Roche e801 platform.

**Findings:**

A total of 2340 individuals participated in the study. SARS-CoV-2 antibody results were available for 70·1% (620/885) and 73·1% (1530/2093) of eligible participants in 2020 and 2021. The median age was 22 (IQR 10-37) years and 978 (45·5%) were men. SARS-CoV-2 seroprevalence was 19·0% (95% CI 15·1-23·5%) in 2020 and 53·0% (95% CI 49·6-56·4) in 2021. The prevalence ratio was 2·47 (95% CI 1·94-3·15) comparing 2020 with 2021 after adjusting for age, sex, and community. Almost half of all participants who tested positive reported no symptoms in the preceding six months.

**Interpretation:**

Following the second wave, one in two people had been infected with SARS-CoV-2 suggesting high levels of community transmission. Our results suggest that 184,800 (172,900-196,700) SARS-CoV-2 infections occurred in these three communities alone, greatly exceeding the reported number of cases for the whole city. Further seroprevalence surveys are needed to understand transmission during the current third wave despite high prevalence of past infections.

**Funding:**

GCRF, Government of Canada, Wellcome Trust, Bavarian State Ministry of Sciences, Research, and the Arts

**Research in context**

**Evidence before this study**

Many national and subnational population-based SARS-CoV-2 seroprevalence surveys have been conducted globally and summarised in a recently published systematic review. Pooled estimates of SARS-CoV-2 seroprevalence in the general population varied greatly by WHO region with 19·6% (95% CI 5·5-33·6, 4 studies), 6·8% (95%CI 5·0-8·5, 13 studies), 4·7% (95%CI 3·6-5·9, 14 studies) testing positive for SARS-CoV-2 antibodies in the South East Asian, American and European regions respectively. Overall the pooled estimated ratio of serologically detected infections to confirmed cases of COVID-19 was 11·1 (95%CI 8·3-14·9) suggesting that for each virologically confirmed SARS-CoV-2 infection, at least ten infections remained undetected by surveillance systems globally. Only two population-based studies from Africa contributed to the systematic review reporting a SARS-CoV-2 prevalence of 8·8% in Ethiopia in April 2020 and 25·4% in Nigeria in August 2020. Both studies had relatively small sample sizes and used rapid antibody tests. We searched PubMed up to June 19, 2021, for peer-reviewed and preprints using the search terms “COVID-19” OR “SARS-CoV-2” AND “prevalence” AND “Africa”. Additionally, we searched bibliographies of identified studies, a database of seroprevalence studies maintained by WHO, and the Google search engine for manuscripts and non-peer reviewed pre-prints. We identified three more studies conducted in Africa reporting SARS-CoV-2 seroprevalence estimates for the general population. Prevalence was 2·1% in Zambia in July 2020, 38·5% in South Sudan in August-September 2020 and 26% in rural and 41% in urban South Africa in March 2021. The South African study was a cohort study and estimated the prevalence following the first and second SARS-CoV-2 wave.

**Added value of this study**

To our knowledge, this is the first population-based SARS-CoV-2 prevalence study done in a low-income country in sub-Saharan Africa, to estimate cumulative SARS-CoV-2 prevalence after the second wave. Despite lower than predicted hospitalisations and deaths due to COVID-19 in Zimbabwe, SARS-CoV-2 seroprevalence in the population is extremely high across age-groups. Seroprevalence more than doubled during the second wave.

**Implications of all the available evidence**

This study demonstrates that laboratory-confirmed case notifications grossly underestimate the true number of infections in Zimbabwe. The majority of SARS-CoV-2 infections were asymptomatic. These findings are crucially important for future SARS-CoV-2 control measures and more specifically for vaccination strategies. If the majority of the population has some immunity due to past infection, a single-dose vaccination approach may be an option. Longitudinal studies in low-income settings in rural and urban populations are urgently needed to understand optimal vaccination strategies in the context of prior infection.

Alt-text: Unlabelled box

## 1 Introduction

**Figure 1:**
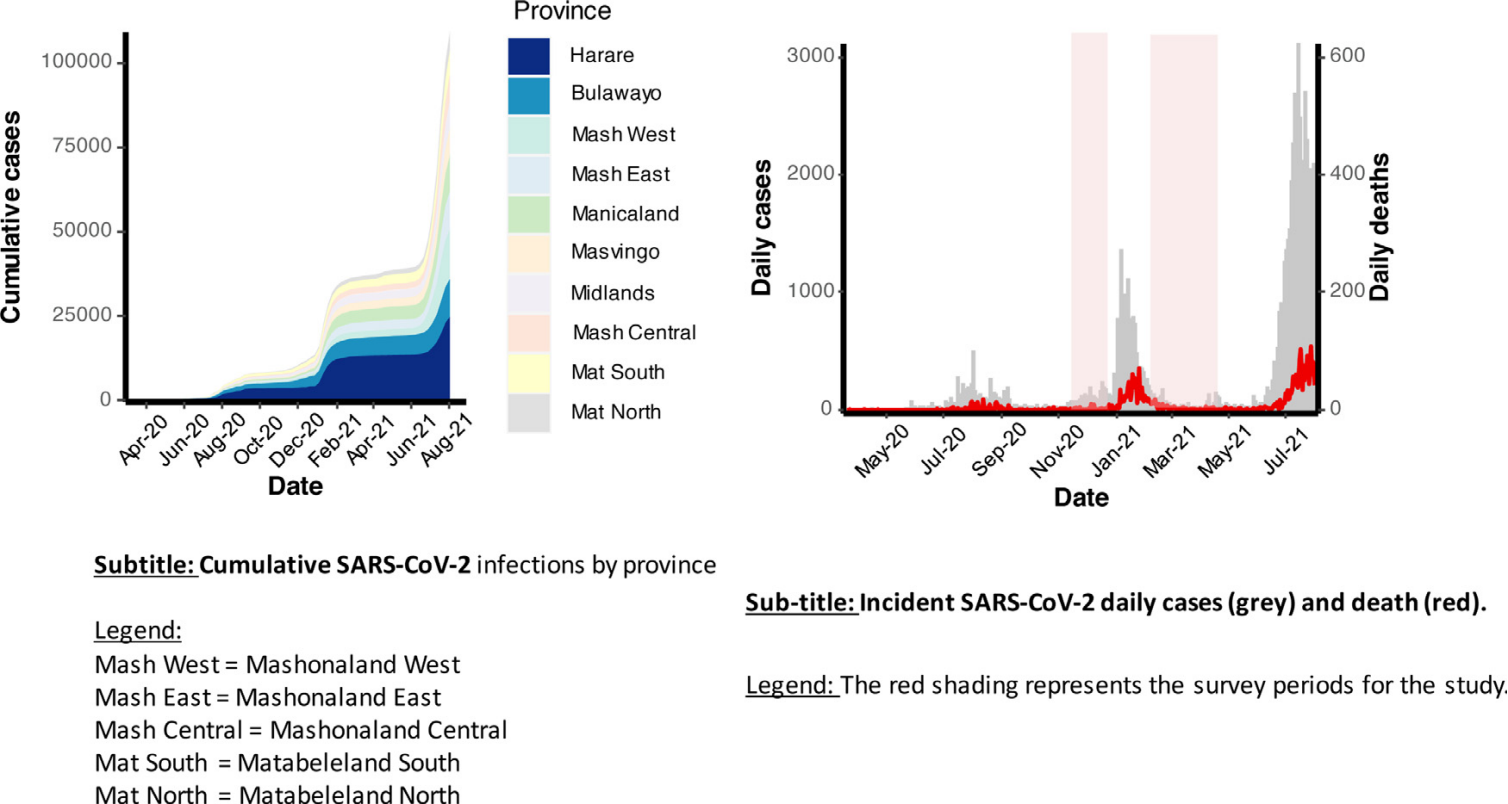
National laboratory confirmed SARS-CoV-2 incident infections and deaths and cumulative laboratory confirmed SARS-CoV-2 infections by province. a Incident SARS-CoV-2 daily cases (grey) and death (red). The red shading represents the survey periods for the study. b: Cumulative SARS-CoV-2 infections by province. Mash West = Mashonaland West; Mash East = Mashonaland East; Mash Central = Mashonaland Central; Mat South = Matabeleland South; Mat North = Matabeleland North.

The World Health Organization (WHO) declared the SARS-CoV-2 outbreak a global pandemic on March 11 2020, following the identification of a cluster of cases of pneumonia, later termed COVID-19, caused by a novel coronavirus (SARS-CoV-2), in Wuhan China in December 2019 [1]. The first case of COVID-19 in Zimbabwe was confirmed on March 20 2020 in a resident who had returned from the United Kingdom [2]. Zimbabwe has had two waves of infections with peaks in early August 2020 and early January 2021 and is currently experiencing a third wave (Figure 1).

A nationwide lockdown was first implemented on March 30 2020, confining people to their homes, and restricting movement to a 5 km radius. All air and land borders were closed to foreign nationals, only allowing returning residents and nationals to enter the country. Use of face coverings and social distancing was mandated, and all public transport, apart from essential state worker transport (e.g. health care workers), was suspended. The lockdown was extended until May 17 2020 [2]. A second national lockdown came into force on January 5 until February 19 2021. The lockdown introduced a 6am – 6pm curfew and a ban on public gatherings and intercity travel with the exception of essential travel including commercial cargo. The second wave in Zimbabwe was associated with the emergence of a new SARS-CoV-2 variant B.1.351 (beta variant) in South Africa [3], which was subsequently also detected in Zimbabwe [4]. Prior to the ongoing third wave, a traveller returning from India was found to have a SARS-CoV-2 infection with the delta variant (B.1.617.2), which is thought to be more transmissible and likely driving the current third wave in Zimbabwe [5].

By July 27 2021 Zimbabwe has reported a total of 101,711 confirmed SARS-CoV-2 infections and 3280 deaths (Figure 1) [6]. Most cases and deaths were reported in Harare and Bulawayo, the two largest cities in Zimbabwe. However, case notification data hugely underestimate the true number of SARS-CoV-2 infections globally [7,8]. Many cases may not be identified due to mild or absent symptoms and/or reluctance to access care or testing [9,10]. In addition, in Zimbabwe, where SARS-CoV-2 testing capacity is limited and population surveillance is minimal, the gap between the number of reported cases and the true number of infection is likely to be even larger [11].

Little information is available about the prevalence of SARS-CoV-2 in the general population in Africa [8]. Community-based surveys from 2020 reported an estimated seroprevalence of 8·8% in Addis Ababa in Ethiopia (301 randomly selected people in April 2020) [12], 2·1% in Zambia (cross-sectional cluster sample survey of households in 6 districts in July 2020) [13] and 25·4% in Niger State in Nigeria (185 randomly sampled people across state in August 2020) [14]. A longitudinal SARS-CoV-2 seroprevalence survey among the general population in rural (Mpumalanga Province) and urban communities (North West Province) in South Africa conducted between July 2020 and March 2021 revealed a prevalence of 7% in rural and 27% in urban communities after the first wave of SARS-CoV-2 infections and 26% in rural and 41% in urban communities after the second wave [15].

Differences in population demographics, living conditions, in-country mobility, risk of virus importation, disease epidemiology, health system access and climate may affect SARS-CoV-2 epidemiology. Large representative population-based seroprevalence surveys are needed to understand the epidemiology of SARS-CoV-2 in Africa and estimate infection rates, calculate infection-hospitalisation and infection-fatality ratios and compare infection burden between waves. These data are crucial to guide public health responses including vaccination strategies.

We report a population-based household SARS-CoV-2 seroprevalence survey conducted in three high-density communities in Harare, Zimbabwe between November 2020 and April 2021. We also compared seroprevalence before (November – December 2020) and after (February – April 2021) the second wave.

## 2 Methods

### 2.1 Study design and study population

We conducted a cross-sectional study on randomly selected households aiming for a total of 2000 participants in three communities in Harare namely Budiriro, Highfield and Mbare, the largest and most densely populated communities in the city.

A sample size of 2000, assuming a design effect of 2 for the variance inflation due to clustering at household level and community level, was calculated to provide adequate precision around a prevalence of 5% (95% CI 3.6-6.4%, precision 1.4%, relative error 28%). Antibody prevalence was not expected to be lower than 5% in the population.

Fieldwork started on November 20 2020 and ended on April 17 2021 with a five-week hiatus due to a nationwide lockdown in January 2021 (Figure 1). In Budiriro, survey rounds were conducted before the second wave (November 20 – December 12 2020) and after (March 23 – April 17 2021), with each round aiming to enrol approximately 500 participants. In Highfield one third of the households were enrolled before the second wave (December 15 – 20 2020) and two thirds after (February 10 - 23 2021). Fieldwork in Mbare was conducted between February 26 and March 21, 2021.

Households were selected through randomly generated GPS coordinates using the QGIS software (version 3.12). All GPS points were loaded onto the application ‘Maps.Me’ for Android (www.maps.me) on the researcher's tablet. Upon arrival the plot closest to the GPS point was selected for the survey. If on a plot there were multiple households living in a house, or multiple houses or flats all households were numbered, and a random number generator (Random Number Generator Plus by Random Apps Inc.) was used to randomly select one household.

Individuals of any age who had slept three out of the last seven nights in the household before the survey team visited were eligible for participation in the survey. Written informed consent was obtained from adults and emancipated minors, parental consent was obtained for participants aged 17 years and younger, and assent was obtained from participants aged 7–17 years, before the study. Ethical approval for the study was obtained from the Medical Research Council of Zimbabwe, the Institutional Review Board of the Biomedical Research and Training Institute, and the London School of Hygiene and Tropical Medicine ethics committee.

### 2.2 Procedures

For each household, a household-level questionnaire was administered including information on the number, age and sex of people living in the household, the number of bedrooms, availability of running water and soap. For households visited after the second wave (from February 2021 onwards), information about any visitors staying in the household overnight and whether or not the visitor had recently travelled to Zimbabwe from outside the country was also collected.

For each participant, an individual-level questionnaire was administered that included information about demographics, occupational status, medical history, SARS-CoV-2 exposures, and history of any symptoms of viral diseases over the past 6 months. From February 2021 onwards questions about travel and sleepovers in other households, as well as attendance at large events (>50 people) were added. All questionnaires were administered using electronic data capture software (SurveyCTO, https://www.surveycto.com/). Dried blood spot (DBS) samples were obtained from all consenting participants using finger prick technique onto a filter card. If household members were not present at the initial visit, then the research team arranged two further follow up visits to enrol all members of the household. If after three visits a household member was still not present, they were not included.

### 2.3 Laboratory testing

DBS samples were thoroughly dried overnight, individually packed in desiccant containing zip-lock bags and stored at -20°C. Shipping to the Department of Infectious Diseases and Tropical Medicine of the Ludwig Maximillian University Munich was performed at room temperature. DBS samples were eluted, and anti SARS-CoV-2 nucleocapsid antibodies were measured as described elsewhere [16]. In brief, DBS cards were punched using a Panthera-PuncherTM 9 Instrument (PerkinElmer). Three discs with 3·2 mm diameter per DBS spot were automatically dispensed into each well of barcoded 96-well plates. Using two JANUS® G3 workstations (PerkinElmer), or adjustable tip spacing multichannel pipettes (Integra Voyager), elution buffer was added. Elution was performed in temperature-controlled shakers (MIUlab ES-60E) for 1h at 37°C with 300 rpm. A total of 80 μl of PBS buffer containing albumin (5%), ammonium thiocyanate (2·5 mM) and Tween 20 (0·5%), was used to elute antibodies off the DBS and suppress background. Samples were transferred to Roche rack packs containing Roche 13/16 micro sample cups (05085713001) and measured in a Roche e801 machine, using improved cut-off values [17]. All positive samples were re-tested to confirm positivity and exclude sample mix ups or other mistakes. A random sample of 10% of negative samples were also retested. The assay has a sensitivity of 99.20% and specificity of 98.65% [16].

### 2.4 Statistical analysis

Statistical analysis was performed using Stata 15.1 (StatCorp, Texas, USA). Prevalence was calculated as number of positive tests divided by number of samples tested, per district and by year (representing before and after the second wave). Age was categorised in 10-year bands for those aged 10+ years and 5-year bands for those under 10. Non-response weights were calculated using age band and sex from the sampling frame of all eligible individuals at participating households, including those who refused to participate or were absent. Separately we calculated prevalence by year adjusting for sensitivity and specificity of the assay [18]. Generalised linear models were used to calculate prevalence ratios using Poisson regression with a log link function, adjusting for age band and community. We adjusted for household level sampling to account for clustering within households.

### 2.5 Role of funding source

The funders had no role in study design, data collection and analysis, decision to publish, or preparation of the manuscript. AF, VS, TB, NR, RAF and KK had access to the data. The decision to submit the manuscript for publication was a joined decision taken by all authors.

## 3 Results

**Figure 2:**
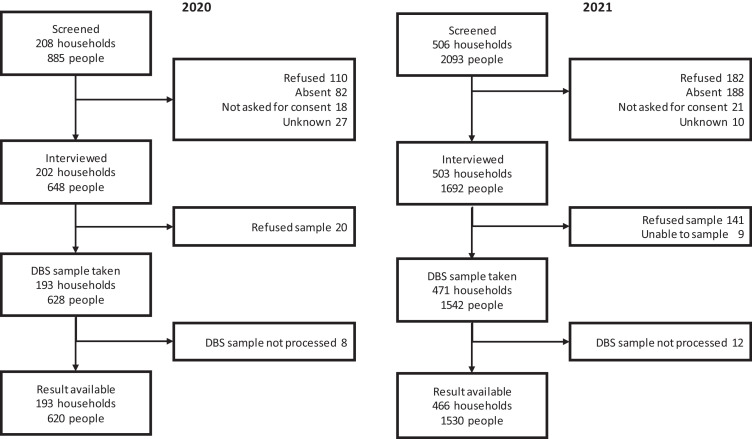
Flowchart of households and participants

Between November 20 and December 20, 2020 (Figure 1), we approached 208 households and enrolled 202 (97·1%) with at least one DBS sample collected in 193 (92·8%). There were 885 potentially eligible participants in the enrolled households. Of the eligible participants 648 (73·2%) were consented and interviewed, 628 (71·0%) provided a DBS sample and 620 DBS samples (70·1%) were of high enough quality to be tested (Figure 2). Between February 10 and April 17, 2021, 506 households were approached and 503 (96·6%) enrolled. From a total of 2093 potentially eligible participants, 1692 (80·8%) were enrolled. DBS samples were processed for 1530 (73·1) participants from 466 (92·0%) households. Survey participation was higher among those aged <20 years (73·5%) and ≥50 years (81·1%) compared to those aged 21-49 years (68·8%) and was higher in women than men (78·2% vs. 65·0%).

In both years, the study included more children and young adults than older individuals (Table 1). The median age of participants was 22 (IQR 10-37) years and the reported HIV prevalence was 8·9% (95% CI 7·5-10·5%) in those aged >15 years. The majority of those aged >15 years were unemployed or self-employed, including street vendors.

**Table 1:** Participants’ characteristics in 2020 and 2021

Characteristics		Survey in 2020		Survey in 2021	
		N	% (95% CI)	N	% (95% CI)
			*N=620*		*N=1530*
**Suburb**	Highfield	160	22·6 (16·6, 29·9)	369	22·2 (18·2, 26·8)
	Budiriro	460	77·4 (70·1, 83·4)	546	38·3 (33·4, 43·5)
	Mbare	0	0	615	39·5 (34·5, 44·7)
**Sex**	Male	259	45·6 (41·7, 49·4)	623	45·5 (43·2, 47·9)
	Female	361	54·5 (50·5, 58·2)	907	54·5 (52·1, 56·8)
**Age**	0-4	68	13·1 (10·5, 16·2)	125	8·6 (7·4, 10·4)
	5-9	89	13·1 (11·6, 16·1)	200	11·9 (10·5, 13·4)
	10-19	133	20·9 (17·9, 24·2)	325	20·5 (18·5, 22·6)
	20-29	106	16·2 (13·1, 19·8)	292	20·3 (18·1, 22·6)
	30-39	105	16·5 (13·8, 19·6)	223	15·5 (13·7, 17·5)
	40-49	69	11·9 (9·4, 14·9)	148	10·9 (9·3, 12·6)
	50-59	22	3·4 (2·2, 5·3)	111	6·4 (5·2, 7·7)
	60-69	20	3·4 (2·1, 5·5)	67	3·7 (2·9, 4·7)
	70-100	8	1·0 (0·5, 2·2)	39	2·3 (1·6, 3·2)
**Household size**	Median (IQR)	-	4 (3-5)	-	4 (3-5)
**Education (missing for 23)**	None	76	13·1 (10·7, 16·0)	149	9·5 (8·2, 11·0)
	Primary	171	28·6 (24·9, 32·6)	380	23·8 (21·7, 25·9)
	Secondary	299	48·0 (44·0, 52·1)	868	57·9 (55·3, 60·4)
	Diploma	33	5·6 (3·9, 8·0)	57	4·1 (3·1, 5·3)
	University	28	4·6 (3·0, 7·1)	66	4·8 (3·6, 6·3)
**Employment status if aged ≥16 (missing for 29)**	Student	51	13·9 (10·7, 17·9)	112	11·0 (9·0, 13·3)
	Formally employed	76	22·3 (17·8, 27·4)	194	21·0 (18·2, 24·1)
	Street vendor	47	12·7 (9·6, 16·5)	147	14·2 (11·9, 16·7)
	Other self-employed	67	19·1 (15·1, 24·0)	143	15·7 (13·0, 18·7)
	Unemployed	125	32·1 (27·3, 37·2)	403	38·2 (34·9, 41·5)
**Primary mode of transport if aged ≥16 (missing for 36)**	Private	59	17·5 (13·4, 22·6)	177	18·1 (15·4, 21·3)
	Public	238	66·2 (59·9, 72·0)	558	55·7 (51·9, 59·5)
	Walking	58	16·2 (12·0, 21·6)	268	26·1 (22·9, 29·6)
**Does the household have running water from the tap?**	Almost never/no	263	43·9 (36·0, 52·1)	300	20·8 (16·8, 25·3)
	Intermittently	222	36·1 (28·7, 44·2)	617	39·9 (34·9, 45·1)
	All the time	135	20·0 (14·4, 27·1)	613	39·3 (34·4, 44·5)
**Medical condition (missing for 23)**	Hypertension	26	4·0 (2·7, 5·9)	102	6·0 (4·9, 7·4)
	Diabetes	7	1·1 (0·5, 2·3)	26	1·6 (1·1, 2·4)
	HIV	20	3·4 (2·1, 5·4)	116	7·2 (5·9, 8·9)
**Ever had a SARS-CoV-2 positive PCR? (missing for 23)**	Yes	22	4·0 (2·5, 6·4)	96	6·8 (5·4, 8·4)
**Close contact (<1m) with laboratory confirmed SARS-CoV-2 case? (missing for 23)**	Yes	15	2·4 (1·2, 4·7)	96	6·8 (5·1, 9·0)
**Any deaths in the household within the previous 6 months**	Yes	48	7·5 (4·3, 12·6)	98	6·3 (4·3, 9·2)

Percentages and confidence intervals are weighted for non-response

**Figure 3:**
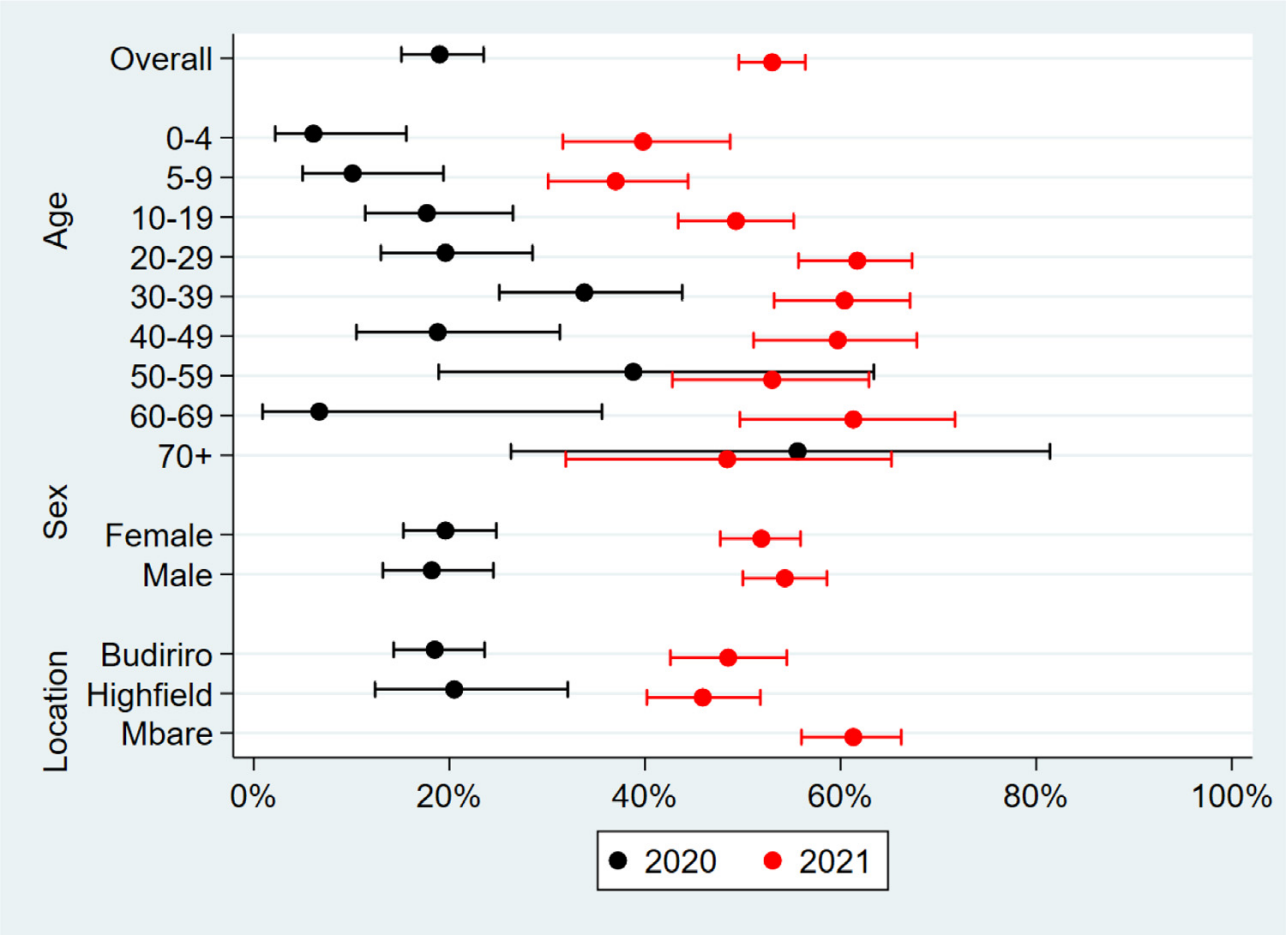
Prevalence of SARS-CoV-2 and 95% CIs by year, age group, sex and suburb. Black = 2020, red = 2021

In 2020, 19·0% (95% CI 15·1-23·5%) of participants were SARS-CoV-2 positive and in February-April 2021 the SARS-CoV-2 prevalence was 53·0% (95% CI 49·6-56·4). The prevalence ratio was 2·48 (95% CI 1·94-3·16) comparing 2020 with 2021 after adjusting for age, sex and community (Table 2). Those less than <20 years of age had a 0·69 (95% CI 0·62-0·77) times lower prevalence than those aged 20-59 years. In the 2021 data, the prevalence ratio for Mbare compared to the other two communities combined was 1.28 (95% CI 1.13-1.44) after adjusting for age and sex (Figure 3). After adjusting for sensitivity and specificity of the assay, the prevalence of SARS-CoV-2 antibodies was 18·0% (95% CI 14·1-22·1%) in 2020 and 52·8% (95% CI 49·3-55·0) in 2021.

**Table 2:** Weighted prevalence of SARS-CoV-2 antibody positivity

Characteristics		Prevalence (95% CI)	Adjusted prevalence ratio (95% CI)	p-value
**Age**	<20	33·4 (29·6, 37·4)	0·69 (0·62, 0·77)	<0·001
	20-59	50·4 (46·8, 53·9)	1	
	60+	47·0 (37·9, 56·2)	0·89 (0·74, 1·06)	
**Sex**	Male	43·6 (39·7, 47·5)	1	0·39
	Female	42·3 (38·9, 45·9)	0·96 (0·88, 1·05)	
**Year**	2020	19·0 (15·1, 23·5)	1	<0·001
	2021	53·0 (49·6, 56·4)	2·48 (1·94, 3·16)	
**Location**	Budiriro	34·7 (30·5, 39·2)	1	<0·001
	Mbare	61·2 (56·0, 66·2)	1·26 (1·09, 1·46)	
	Highfield	38·4 (33·1, 44·0)	0·97 (0·82, 1·16)

Table 3 shows characteristics associated with SARS-CoV-2 seropositivity in the 2021 survey round, adjusted for age and community. People who had attended a large event of more than 50 people, such as weddings, funerals, or religious services, in December 2020-January 2021 were 13·7% (95% CI 1·0-28·6%) more likely to be SARS-CoV-2 positive, and those who reported close contact with a confirmed SARS-CoV-2 case were 20·9% (95% CI 3·2-41·6%) more likely to be positive, adjusting for age, location and non-response weighting. There was no association between SARS-CoV-2 prevalence and sex, education, comorbidities, staying away from home overnight, having a confirmed SARS-CoV-2 case in the household, or availability of running water or soap (Table 3). For the two suburbs that were sampled in both years, the adjusted prevalence difference comparing the periods before and after the second wave was 27·0% (95% CI 22·5-31·6). This represents the cumulative incidence between the two waves. Table 4 shows the prevalence of reported symptoms by SARS-CoV-2 antibody status. Almost half of participants testing positive for SARS-CoV-2 antibodies (49·7%) reported no symptoms within the previous six months.

**Table 3:** Generalised linear model of factors associated with COVID antibody positivity in 2021, adjusting for household level sampling

Characteristics		Weighted prevalence (95% CI)	Prevalence ratio (95% CI)^a^	p-value
**Age**	0-4	39·8 (31·6, 48·7)	1	
	5-9	37·0 (30·1, 44·4)	0·94 (1·09, 1·54)	
	10-19	49·3 (43·4, 55·2)	1·24 (0·99, 1·54)	<0·001
	20-29	61·7 (55·7, 67·3)	1·54 (1·23, 1·90)	
	30-39	60·4 (53·3, 67·1)	1·52 (1·21, 1·90)	
	40-49	59·7 (51·1, 67·8)	1·51 (1·18, 1·92)	
	50-59	53·0 (42·8, 62·9)	1·33 (0·99, 1·74)	
	60-69	61·3 (49·7, 71·7)	1·51 (1·16, 1·97)	
	70-100	47·3 (31·1, 64·0)	1·15 (0·76, 1·79)	
**Location**	Budiriro	48·6 (42·6, 54·5)	1	<0·001
	Mbare	61·2 (56·0, 66·2)	1·25 (1·08, 1·45)	
	Highfield	46·1 (40·3, 51·9)	0·94 (0·79, 1·12	
**Sex**	Male	54·3 (49·9, 58·6)	1	0·25
	Female	51·8 (47·9, 56·0)	0·95 (0·86, 1·04)	
**Education (missing for 23)**	None	39·6 (31·9, 47·8)	1	0·42
	Primary	43·8 (38·1, 49·6)	1·03 (0·71, 1·50)	
	Secondary	57·4 (53·5, 61·2)	1·13 (0·75, 1·69)	
	Diploma	69·2 (54·4, 80·9)	1·32 (0·85, 2·05)	
	University	60·6 (46·6, 73·0)	1·18 (0·74, 1·89)	
**Any comorbidity (HIV, hypertension or diabetes) (missing for 23)**	Yes	52·7 (49·1, 56·3)	0·92 (0·79, 1·08)	0·31
	No	55·7 (48·6, 62·5)	1	
**HIV (missing for 23)**	Positive	55·8 (46·0, 65·2)	0·91 (0·76, 1·09)	0·31
	Negative	52·9 49·4, 56·3)	1	
**Diabetes (missing for 23)**	Yes	55·6 (35·4, 74·1)	0·97 (0·69, 1·37)	0·88
	No	53·0 (49·6, 56·4)	1	
**Hypertension (missing for 23)**	Yes	58·0 (47·9, 67·6)	1·02 (0·84, 1·23)	0·85
	No	52·8 (49·3, 56·2)	1	
**Attended a large event between Dec 2020-Jan 2021**	Yes	61·6 (55·1-67·6)	1·14 (1·01, 1·29)	0·037
	No	51·0 (47·2-54·8)	1	
**Stayed away from home overnight between Dec 2020-Jan 2021**	Yes	57·1 (51·3-62·7)	1·06 (0·94, 1·19)	0·35
	No	52·1 (48·2-55·9)	1	
**Confirmed COVID-19 in household since Dec 2020**	Yes	57·4 (41·1, 72·2)	1·00 (0·76, 1·30)	0·98
	No	53·2 (49·7, 56·6)	1	
**Overnight visitor to the household between Dec 2020-Jan 2021**	Yes	57·0 (50·6, 63·1)	1·06 (0·93, 1·21)	0·36
	No	51·8 (47·8, 55·9)	1	
**Close contact with a confirmed COVID-19 case**	Yes	67·9 (57·3, 76·9)	1·21 (1·03, 1·42)	0·017
	No	52·0 (48·5, 55·5)	1	
**Household has running water coming from the taps**	Almost never	51·4 (43·4, 59·2)	1·03 (0·83, 1·27)	0·67
	Intermittently	51·2 (46·1, 56·2)	0·95 (0·83, 1·09)	
	All the time	55·8 (50·2, 61·1)	1	
**Soap or sanitiser readily available in the household**	Almost never	59·8 (34·2, 81·0)	1·18 (0·75, 1·84)	0·35
	Intermittently	48·6 (41·2, 56·0)	0·90 (0·76, 1·06)	
	All the time	53·8 (50·0, 57·6)	1	

a adjusted for age band and location

**Table 4:** Reported symptoms in the previous 6 months by SARS-CoV-2 antibody status

Symptom	Negative, n (%)	Positive, n (%)
*N*	*1208*	*919*
Cough	232 (19·2)	251 (27·3)
Cold	363 (30·1)	370 (40·3)
Fever	117 (9·7)	151 (16·4)
No sense of smell	42 (3·5)	71 (7·7)
Shortness of breath	32 (2·7)	61 (6·6)
Any symptoms	468 (38·7)	462 (50·3)

*excluding 23 tests with no symptoms questionnaire (17 negative, 6 positive)

## 4 Discussion

According to our data one in two people (53.0%, 95% CI 49·6-56·4%) had been infected with SARS-CoV-2 by April 2021 in three high density communities in Harare, Zimbabwe. The seroprevalence survey results suggest that 184,800 (172,900-196,700) SARS-CoV-2 infections occurred in these three communities alone, which greatly exceeds the reported number of cases for the whole city (13,044 on April 19 2021). Unfortunately, notified SARS-CoV-2 infections are only reported by province and notifications by district or subdistrict are not available. Hence direct comparisons between the number of infections estimated from the seroprevalence survey and notification data are difficult. The three communities have an estimated population of 350,000, which accounts for 19% of the population in the capital [19,20]. If seroprevalence was similar across Harare, almost 1 million (972,631) SARS-CoV-2 infections would have occurred during the first and second wave. Thus the ratio of serologically detected infections to confirmed SARS-CoV-2 cases in Harare is likely to be much higher than that reported from South Africa [15] (18·0-27·1) but similar to data from Juba, South Sudan with an estimated ratio of more than 100 [21]. The cumulative incidence estimate of 27.0% suggest that 68,500 new SARS-CoV-2 infections occurred during the second wave in Budriro and Highfield alone, while the number of infections reported in Harare during the second wave was only 8700.

Our data confirm that there has been extensive community transmission during the first and second wave. The data from two communities in Harare showed that 18·0% (95% CI 14·1-22·1%) of the population had been infected with SARS-CoV-2 following the first wave. The prevalence of past infection was highest among the 30–39 year age-group with no difference between men and women, which is comparable to other data from sub-Saharan Africa [14,15,21]. SARS-CoV-2 seroprevalence more than doubled during the second wave, confirming intense community transmission. Factors that may have contributed to high levels of community transmission especially during the second wave are more interprovincial and international travel as restrictions were being eased in December 2020, a national public holiday (Christmas) resulting in inter-generational mixing and travelling to and from rural villages for family reunions, and the emergence of a more transmissible SARS-CoV-2 variant B.1.351 (beta variant) in South Africa [3]. This was also reflected in the higher number of laboratory confirmed SARS-CoV-2 infections and deaths during the second wave compared to the first wave [22].

Following the second wave, SARS-CoV-2 seroprevalence was higher in the population of Mbare compared to the prevalence in the other two communities. Mbare is the oldest high-density community in Harare, established in 1907, and home to a major trading market (Mbare Musika) and bus station for intercity transport. In contrast to the other two communities, Mbare has many blocks of flats built in the 1940s as “hostels”. The capacity of those flats is greatly surpassed with occupancy rates for single rooms sometimes going up to 2-3 households [23]. The proximity of Mbare to the central business district of Harare, the working and trading opportunities provided by Mbare Musika and the transport hub results in movement in and out of Mbare from the other provinces. This may explain the higher seroprevalence in Mbare compared to the other two communities.

The proportion of individuals reporting previous symptoms of flu-like illness was higher among those with positive SARS-CoV-2 serology compared to those with a negative serology. However, almost half of those with positive serology did not remember any symptoms in the 6 months prior to the survey. This is in line with a high proportion of asymptomatic or mildly symptomatic SARS-CoV-2 infections especially among young people [9,24,25], but may also be explained by people failing to remember any symptoms. Few people reported a PCR-confirmed SARS-CoV-2 infection in the past. No difference in the proportion of seropositivity between people diagnosed with HIV, hypertension and/or diabetes was observed. Although individuals with co-morbidities are more likely to require hospital admission, their risk of getting infected is likely comparable to those without co-morbidities.

Those with a positive serology were more likely to have had contact with a person with confirmed SARS-CoV-2 infection and attended large events with more than 50 people – even though most of these events took place outdoors. Most households had at least intermittent access to soap and/or hand sanitisers and masks, while access to running water was available in Highfield and Mbare only.

The strengths of our survey include the relatively large sample size and high household participation rates. Furthermore we used a testing methodology which has been rigorously validated on thousands of paired serum and DBS samples [17]. All samples testing positive underwent a repeat test and a random sample of negative samples were retested.

Our study has several limitations. While we recruited more than 95% of approached households, we only obtained DBS samples from 72·2% of potentially eligible study participants. Reasons for not being able to obtain a DBS sample was refusal by the study participant or absenteeism. Similar to other surveys, men aged 20-49 years were less likely to be included in the survey as they were away from the households for work [15,21]. However, we accounted for that in the overall seroprevalence estimate. We relied on self-reporting of laboratory confirmed SARS-CoV-2 infection, HIV, diabetes and hypertension which may be unreliable and subject to underreporting due to stigma. We did not collect data on socio-economic status. We estimated the cumulative SARS-CoV-2 incidence during the second wave by comparing results of two cross-sectional surveys. A cohort design may have been more appropriate, but subject to attrition bias. The seroprevalence survey was restricted to three communities in Harare and hence the results are not generalisable to other more rural settings, where transmission may have been less intense. Also the seroprevalence survey was restarted on February 10 2021 at a time when SARS-CoV-2 notifications had just decreased to pre-wave levels. Hence we may have underestimated seroprevalence post second wave in Highfield because some individuals with recent or current infection may not yet have seroconverted. Seroprevalence may have been underestimated due to antibody decay over time [26].

In conclusion, we found evidence of an extremely high rate of SARS-CoV-2 infections in three high density communities in Harare following the second SARS-CoV-2 infection wave. Prevention and control measures such as contact restrictions (lockdowns and school closures), social distancing, heightened hygiene measures and improved case detection were either not sufficiently timely (i.e. too late) or effective in limiting transmission. In Mbare, the proportion of individuals with evidence of past infection was more than 60%, which is close to the community infection threshold. However, new variants and waning immunity may mean that re-infection and onward transmission of individuals previously infected is possible.

Also, other more rural parts of the country are unlikely to have equally high rates of past SARS-CoV-2 infections. The third wave which seems to be disproportionally affecting the Mashonaland West province may be explained by that [18]. Ongoing seroprevalence combined with molecular infection surveys across different settings are important to inform vaccination strategies and future public health response.

## Funding

GCRF, Government of Canada, Wellcome Trust, Bavarian State Ministry of Sciences, Research, and the Arts

## Contributors

KK, RAF, CN, HM, SR, MH conceptualised the study. AF, TB, NR, IDO collected the data in Zimbabwe. PC and KM supported field work and communication within the communities. VS lead the data analysis with input from TB and NR. AW and RRA established and validated the assay. AW oversaw laboratory testing. RRA and IP conducted the laboratory test. KK, VS, AF and RAF wrote the first draft of the manuscript. All authors provided input to the draft manuscript and read and approved the final manuscript.

## Data sharing statement

Individual, anonymised participant data and a data dictionary will be available through The London School of Hygiene & Tropical Medicine repository (Data Compass) 12 months after publication of results.

## Declaration of Competing Interest

AW declared that Roche provided reagents and machine for discounted rates for all projects involving SARS-CoV-2 serology. AW holds part of a patent application with the German Patent and Trade Mark Office relating to safe transport of DBS for analytical purpose; this application was not used in the current study. RAF received funding from the Wellcome Trust since 2017, is a member of the Zimbabwe national COVID19 Advisory Panel and a member of the CDC Africa Strategic Advisory Group of Experts for COVID19. All the other authors report no conflicts.
